# Associations of nerve conduction parameters and OCT angiography results in adolescents with type 1 diabetes

**DOI:** 10.1371/journal.pone.0252588

**Published:** 2021-06-04

**Authors:** Marta Wysocka-Mincewicz, Joanna Gołębiewska, Marta Baszyńska-Wilk, Andrzej Olechowski, Aleksandra Byczyńska, Maria Mazur, Monika Nowacka-Gotowiec

**Affiliations:** 1 Clinic of Endocrinology and Diabetology, Children’s Memorial Health Institute, Warsaw, Poland; 2 Department of Ophthalmology, Children’s Memorial Health Institute, Warsaw, Poland; 3 Faculty of Medicine, Lazarski University, Warsaw, Poland; 4 Ophthalmology Department, James Cook University Hospital, Middlesbrough, United Kingdom; 5 Electromyography and Evoked Potential Laboratory, Children’s Memorial Health Institute, Warsaw, Poland; Weill Cornell Medicine-Qatar, QATAR

## Abstract

**Aim:**

To evaluate dependence of abnormalities in peripheral nerves and retina in children with type 1 diabetes (T1D) using optical coherence tomography angiography (OCTA) and nerve conduction studies (NCS).

**Material and methods:**

50 adolescents with T1D without any signs and symptoms of diabetic retinopathy and neuropathy (mean age 16.92±1.6 years, diabetes duration 6.88 ±4.34years) were included. In OCTA capillary plexuses superficial (SCP) and deep (DCP) vessel density: whole, foveal and parafoveal, ganglion cell complex (GCC), loss volume focal (FLV) and global loss volume (GLV) were analyzed in relation to NCS parameters (motor nerves median and tibial potential amplitude (CMAP), velocity (CV), distal latency (DML) and F wave and sensory nerves median and sural potential amplitude (SNAP), CV and distal latency (DSL).

**Results:**

We detected the correlations between median sensory SNAP and GCC (r = -0.3, p <0.04), motor nerves tibial DML and CV and FLV (respectively r = -0.53, p<0.001, and r = -0.34, p<0.05), and median DML and GLV (r = 0.47, p<0.001). Vessel densities were related to changes in motor nerves tibial velocity (whole SCP r = 0.43, p <0.01, parafoveal SCP r = 0.41, p <0.01), CMAP (parafoveal SCP r = -0.35, p<0.03), median DML (whole DC r = 0.36, p<0.03, foveal DCP r = 0.37, p<0.02) and in sensory median SNAP (whole SCP r = -0.31, p<0.05).

**Conclusions:**

In adolescents with T1D without diabetic neuropathy and retinopathy we detected associations between NCS and OCT and OCTA parameters, regarding decreased GCC and density of superficial and deep vessel plexuses in relation to DML and CV and amplitudes of sensory and motor potential.

## Introduction

Type 1 diabetes (T1D) in children population is the third most common chronic disease, and its incidence is still increasing. Diabetes is burdened by the risk of micro and macrovascular complications. The most common of the microvascular ones is diabetic retinopathy (DR) and concerns the majority of patients with long-standing type 1 diabetes mellitus [1]. Diabetic neuropathy (DN) is very disabling, and silently developing complication. The lack of effective treatment for DN highlights the importance of early diagnosis to prevent progression. While in children symptomatic late complications of T1D are exceedingly rare, careful examination might reveal disturbances. Pubertal status and the prepubertal duration of diabetes have strong impact on extension of the complication risk [2, 3]. New imaging technologies have created possibility to identify structural changes in retina, in preclinical status. Optical coherence tomography (OCT) proved to be a tool enabling the reproducible and quantitative assessment of the retinal layers, which currently remains the most precise method to measure retinal and choroidal thicknesses *in vivo*. Optical coherence tomography angiography (OCTA) has proven as a non-invasive tool, which enables reproducible, quantitative assessment of the retinal microcirculation and seems to be an effective method in the detection of early microcirculation disorders [4]. The aim of the study was to assess the relationship between potential abnormalities in neuroretina and retinal vessels and peripheral nerves function measured by nerve conduction studies (NCS) in youths with T1D. We hypothesized that OCT- derived parameters could be useful in identifying patients who require appropriate examination of the neuropathic complication of diabetes.

## Materials and methods

It was a prospective, observational study in a group of adolescents with diagnosis of T1D based on International Society for Pediatric and Adolescent Diabetes (ISPAD) criteria, treated with insulin. The patients remain under control of the Clinic of Endocrinology and Diabetology of Children’s Memorial Health Institute. A neurological assessment of adolescents over 15 years has been proposed and performed on all persons under 18 years prior to transfer to an adult clinic. Median, tibial and sural nerve conduction studies were performed in 55 individuals, however in 5 persons tests were recorded in the other nerves, because of technical problems and those data were excluded from the analysis. Finally the data of 50 adolescents were included to the study (demographic data is summarized in Table 1). All children in the study were of European descent. The study was approved by the Bioethics Committee Children’s Memorial Health Institute in Warsaw and followed the tenets of the Declaration of Helsinki. A written informed consent had been obtained from the patient’s legal guardians and from patients over 16 years of age after explanation of the nature of the non-invasive study, before the tests started. Inclusion criteria were previously recognized T1D, not in remission status, no DN and DR, and assent agreement for participation. A modified neuropathy disability score was used to diagnose diabetic peripheral neuropathy (DPN) [5, 6]. The modified neuropathy disability score (NDS) was derived for neurological examination of vibration perception, sharp/blunt sensation, temperature sensation using Neurotip device, and ankle reflexes using a reflex hammer. A score 0 is given to normal response and 1 for abnormal response for each individual test component, for each leg independently (total 10 points). A modified NDS ≥3 indicate on neuropathy, with higher scores indicating more severe disease [5]. Patients with NDS < 3 were included in the study group (all patients had NDS = 0).

**Table 1:** Characteristics of the patients with type 1 diabetes.

Investigative trait	Mean	SD	Median	Min	Max
Age (years)	16.92	1.6	17.00	15	18
Diabetes duration (years)	6.88	4.34	6.6	0.02	15.33
Age at onset (years)	10.00	4.12	10.63	2.3	17.04
Weight (kg)	66.49	12.83	65.00	40.50	93.50
Height (cm)	172.14	9.47	171.50	157	191.50
BMI (kg/m2)	22.4	3.77	22.18	15.81	32.56
HbA1c current (%)	8.68	1.55	8.5	6.4	13.7
HbA1c mean (%)	8.43	1.38	8.2	6.3	12.08

SD = standard deviation; BMI = body mass index; HbA1c = glycated hemoglobin A1c

Exclusion criteria included the history of prematurity, any retinal pathologies, such as hereditary retinal dystrophies, vitreoretinal diseases, myopia or hypermetropia (more than 3 diopters), history of uveitis. All patients underwent a general medical examination. In the study group we did not identify any patients with diabetic retinopathy on fundus ophthalmoscopy. No patient had symptoms and signs of DN and DR. None of the patients was affected by system hypertension and renal dysfunction. Metabolic control was evaluated by glycated hemoglobin A1c (HbA1c) current to the study, and mean value for whole diabetes duration (minimum 4 tests per each year). OCT imaging (AngioVue, Optovue, Fremont, CA) was performed after pupil dilatation (using tropicamide 1.0%). The standard GCC scan protocol, which consists of one horizontal line with a 7 mm scan length and 15 vertical lines with a 7 mm scan length and a 0.5 mm interval centered at 1 mm temporally to the fovea, was used in all the participants. The GCC analysis algorithm calculated automatically the GCC thickness (defined as the distance from the internal limiting membrane to the outer boundary of the inner plexiform layer) and enable the analysis of diffuse and focal GCC defects by calculating global loss volume and focal loss volume. Ganglion cell complex (GCC) focal loss volume (FLV) is an OCT parameter, which describes the percentage of focal ganglion cell complex loss over the entire ganglion cell map. GCC global loss volume (GLV) is a measure of the average ganglion cell complex loss over the entire ganglion cell complex map … All acquired images were inspected, and if automatic segmentation errors occurred or resulted in measurement artifacts, manual segmentation was performed. OCTA examination was performed using the standard macular protocol. All of the eye scans were of a 3 x 3-mm scanning area centered on the fovea. AngioVue automatically segments the area into four layers, including superficial capillary plexus layer (SP), deep capillary plexus layer (DP), outer retinal layer and choriocapillaries. Vessel density is calculated as a percentage of the area containing blood vessels. In the study the following parameters were evaluated: superficial capillary plexus (SCP) and deep capillary plexuses vessel density (DCP): whole, foveal and parafoveal. The scans were taken for both eyes and the data for the right eye was analyzed. Poor quality scans, with motion artifacts or blurred images, were excluded from the analysis. OCT and OCTA results of the studied group were compared with the control group of healthy, age matched subjects (N = 21, mean age 16,2±3), who were children of the hospital staff. The electrophysiological tests were done for all patients by Sierra Summit electromyography machine (Cadwell), which permits the storage of 16 F responses and the automatic calculation of the parameters. NCS were performed with conventional neurolophysiological techniques using surface electrodes. Patients were placed in supine position in relative relaxation, in room temperature between 22–24°C. Extremities were warmed up to a surface temperature between 32–36°C. The simplified NCS protocol for non-symptomatic patients was used. For each patient, the standard nerve conduction measurements were performed on tibial motor and sural sensory nerves in the right lower extremity, and median nerve (motor and sensory) in the right upper extremity. Motor nerve conduction velocity (MCV), compound muscle action potential (CMAP), distal motor latency (DML) were determined in the motor nerves. Sensory nerve conduction velocity (SCV), sensory nerve action potential amplitude (SNAP), and distal sensory latency (DSL) were determined in the sensory nerves (median- orthodromic stimulation; sural- antidromic stimulation). Minimal latencies of F waves were determined for motor nerves (median and tibial). The electrophysiological studies of all the 55 patients were performed by the same physician.

### Statistical analysis

The data was described by mean, median, standard deviation and minimal and maximum values. Values with normal distribution (checked using Shapiro-Wilcs test) were analyzed by Pearson correlation, and those which were not normal in distribution, by Spearman rank R correlations. Comparison of OCT parameters between studied and control groups were performed using T-Student test or U- Mann-Whitney, dependently on normality of distribution. A level p<0.05 was recognized as statistically significant. Tests were performed using TIBCO Software Inc. (2017) Statistica version 13 StatSoft Company.

## Results

The characteristics of the T1D group are summarized in Table 1. The T1D and control groups were not different in OCTA parameters except parafoveal SCP (Table 2). In the study group of youths with T1D, five persons have abnormal NCS in tibial nerve (defined as minimum two parameters above norms) and 1 in sensory median (one nerve in each case).

**Table 2:** Comparison of OCT and OCTA parameters between T1D and control groups.

	T1D group	Control group	p level
GCC (μm)	98.76±6.1	98.28±6.5	p = 0.7
FLV (%)	0.22±0.2	0.25±0.2	p = 0.6
GLV (%)	1.3±1.5	1.9±2	p = 0.2
FAZ (mm^2^)	0.26±0.1	0.26±0.07	p = 0.7
Whole SCP (μm)	51.1±2.2	52.3±2.3	p = 0.1
Foveal SCP (μm)	32.2±4.5	31.2±4.1	p = 0.4
Parafoveal SCP (μm)	52.5±2.5	54.6±2.2	p<0.005
Whole DCP (μm)	58.02±1.7	55.1±1.3	p = 0.1
Foveal DCP (μm)	31.9±4.8	30.1±3.8	p = 0.4
Parafoveal DCP (μm)	60.5± 1.7	61.6±2.5	p = 0.1

GCC- ganglion cell complex, GLV- global loss volume, FLV- focal loss volume, FAZ- foveal avascular zone, SCP -superficial capillary plexus vessel density, DCP- deep capillary plexus vessel density.

We detected correlations between sensory median action potential and GCC (r = -0.3, p <0.04), distal motor tibial latency and velocity and FLV (respectively r = - 0.53, p <0.001, and r = - 0.34, p <0.05) and median DML and GLV (r = 0.47, p <0.001). Vessel densities were related to changes in motor nerve tibial parameters—velocity (whole SCP r = 0.43, p <0.01, parafoveal SCP r = 0.41, p <0.01), and with CMAP (parafoveal SCP r = - 0.35, p <0.03), with sensory median SNAP (whole SCP r = -0.31, p <0.05,), and with median DML (whole DC—r = 0.36, p <0.03, foveal DCP r = 0.37, p <0.02). When we analyzed the dependencies between demographic and metabolic parameters, we observed correlation between diabetes duration and FAZ (r = 0.29, p < 0.05), and sural CV (r = - 0.4, p < 0.003), current HbA1c and whole SCP (r = -0.4, p <0.01), and parafoveal SCP (r = - 0.37, p < 0.02), and mean HbA1c and sural CV (r = - 0.38, p < 0.005). Age at the time of the study correlated with GCC (r = 0.3, p < 0.05), GLV (r = - 0.38, p < 0.01), parafoveal DCP (r = - 0.32, p < 0.05), as well as sural DSL (r = 0.3, p < 0.03), and CV (r = - 0.36, p < 0.01).

## Discussion

In the study group of adolescents (close to maturity) patients with T1D without DR and DPN, we did not observe differences in OCTA results compared to the control group. However, we revealed significant correlations between function of peripheral and quasi peripheral nervous system, as neurons of retina. Various studies reported neural tissue loss, in particular retinal ganglion cells, apoptosis of retinal glial and neural cells and decreased thickness of inner retinal layers before DR becomes clinically detectable [7, 8]. The exact mechanism for inner retinal loss is not clear and some authors investigated the relationship between DN and retinal tissue thickness [9, 10]. There is controversy about the exact mechanism of DN development in patients with T1D. Several papers have identified disturbances in the inner retinal layers of patients with DR or with minimal DR, what suggest that neuroretinopathy could precede microvascular changes [11–13]. However, DN is thought to be characterized by ischemia, and a close link of long-standing diabetes, and both neural and vascular dysfunction has been previously established [14, 15]. Loss of GCC could be a consequence of neuronal damage induced by hyperglycemia or ischemia, oxidative stress, polyol pathway damage or elevated level of glutamate [16]. Most reports reveal that the retina becomes thinner in DR [17, 18]. In our previous study we confirmed these results, as there were significant differences in parafoveal thickness (PFT) between the diabetic and healthy groups [19]. However, the decreased PFT was not related to the decreased GCC thickness in our group of T1D patients. We agree with Srinivasan et al., who hypothesise that microglial changes, which can occur prior to neuronal cell death may be the reason of decreased PFT and thinner parafovea may precede detectable changes in neuroretinal layer thickness [20]. Due to our previous findings, we did not take PFT into analysis now [21]. In our study GCC was significantly and strongly associated with median SNAP. Srinivasan et al. found significantly higher FLV and GLV in patients with diabetic neuropathy compared to those without neuropathy and the control group [20]. Abnormal FLV was observed in a larger part of patients with diabetes complicated by DPN. In our study we observed correlation between FLV and tibial CV and DML. Srinivasan observed enlarged GLV depending on age [20]. Our study group was rather homogenous in age, but we observed age correlations with GCC and GLV. In our previous analysis we did not detect any correlations of GCC, GLV, FLV and age, age at onset and diabetes duration [19]. In our group mean HbA1c correlates only with sural nerve conduction velocity, but not with any of the OCT parameters. We observed significant correlation between current HbA1c and whole and parafoveal SCP. In this study group FAZ correlated with diabetes duration, what was not observed in our previous studies [21]. Electrophysiologic studies conducted by Karsidag et al. in patients with recently diagnosed type 1 diabetes (under 1 year of diabetes duration, poor metabolic controlled, mean HbA1c 9,1%, and all on basal-bolus therapy) revealed abnormal values of 86,7% sural nerves, 83,3% peroneal, 73,3% posterior tibial and 66,7% median nerves [22]. The most frequently abnormal parameter in nerves was distal latency, and F wave latency and conduction velocity. In our group abnormal were only 10% of posterior tibial and 2% of sensory median nerves, and the most frequent parameters were distal latency of tibial nerve and CV of sensory median nerve. Conduction velocity varies with diameter of nerve fiber, thickness of the myelin sheath, distance between Ranvier nodes and length of nerve [23]. Electrodiagnostic parameters are also related to number of axons, extend of demyelination, axonal resistance and could revealed conduction block. Electrophysiologic studies suggested that for DN axonal changes are characteristic, which results predominantly in decreasing of potential amplitude, but also segmental demyelination and remyelination which effect disturbances in conduction velocities and distal latencies in some cases [23]. Lee et al. showed after 5 year observation of children with T1D significant diminishing of CMAP of median, ulnar and posterior tibial nerves, but not in peroneal and SNAP of median and sural sensory nerves but not in ulnar [24]. However, both in our group and in the Karsidag et al. study increase of CMAP and SNAP during the first years of diabetes was observed [22]. Also, in Parano et al. study on population of healthy children potential amplitude of every tested nerves increase proportionally according to age [25]. Potential amplitude is very fragile parameter, which could be easily perturbed. This parameter is influenced by many factors as temperature of skin and room, humidity, patient hydration, and many else. Low temperature of tested extremities decreases conduction velocities and increases amplitude. Therefore, in the study the extremities were warmed up. In our analysis we observed significant correlation between parameters of ganglion cell complex (GCC, FLV and GLV) and parameters of nerve function, especially median and tibial posterior. In this group of adolescents without DN and DR, the most significant was coincidence of changes in median distal motor latency and GLV, and tibial conduction velocity and FLV. Matanovic at al. also detected the most sensitive for glucose fluctuations distal latencies of tibial nerve, even though they tested patients in mean 43 day after diabetes diagnosis [26]. There was previously noticed that the best indicator of neuropathic changes was F-wave latencies, what was not observed in our group.

There are some limitations of the study. This was a single–centre study, involving a limited number of older adolescents. We did not perform corneal confocal microscopy, which would be higher specific comparator for OCT results, but we were interested in associations between the peripheral nervous system and the parameters of the function of the cranial nerve, which is the optic nerve. Our results have indicated the value of OCT and OCTA in monitoring the progress of neurodegeneration in individuals with T1D and can be used as an imaging bioindicator to assess DPN progress or preventive effects of therapies. To our knowledge this is the first such analysis of adolescents with type 1 diabetes. Such diagnostic is particularly important in the detection of subclinical forms of neuropathy, when changes are still mostly reversible and when rapid and good metabolic control can still influence the patient improvements. However, further cross-sectional studies are needed to provide data on the progress of these disorders.

## Supporting information

S1 Data (XLSX)

## References

[1] Girach A, Manner D, Porta M. Diabetic microvascular complications: can patients at risk be identified? A review. Int J Clin Pract. 2006; 60:1471–1483 doi: 10.1111/j.1742-1241.2006.01175.x 17073842

[2] Holl RW, Lang GE, Grabert M, Heinze E, Lang GK, Debatin KM. Diabetic retinopathy in pediatric patients with type-1 diabetes: effect of diabetes duration, prepubertal and pubertal onset of diabetes, and metabolic control. J Pediatr 1998;132:790–794 doi: 10.1016/s0022-3476(98)70305-1 9602187

[3] Cho YH, Craig ME, Donaghue KC. Puberty as an accelerator for diabetes complications. Pediatr Diabetes 2014;15:18–26 doi: 10.1111/pedi.12112 24443957

[4] Jia Y, Tan O, Tokayer J, et al. Split-spectrum amplitude-decorrelation angiography with optical coherence tomography. Opt Express 2012; 20:4710–4725 doi: 10.1364/OE.20.004710 22418228 PMC3381646

[5] Young MJ, Boulton AJ, MacLeod AF, Williams DR, Sonksen PH. A multicentre study of the prevalence of diabetic peripheral neuropathy in the United Kingdom hospital clinic population. Diabetologia 1993; 36:150–154 doi: 10.1007/BF00400697 8458529

[6] Meijer JWG, Smit AJ, Sonderen EV, Groothoff JW, Eisma WH, Links TP. Symptom scoring systems to diagnose distal polyneuropathy in diabetes: the Diabetic Neuropathy Symptom score. Diabetic Medicine 2002; 19:962–965 doi: 10.1046/j.1464-5491.2002.00819.x 12421436

[7] Barber AJ, Lieth E, Khin SA, Antonetti DA, Buchanan AG, Gardner TW. Neural apoptosis in the retina during experimental and human diabetes. Early onset and effect of insulin. J Clin Invest. 1998;102:783–791 doi: 10.1172/JCI2425 9710447 PMC508941

[8] Barber AJ. A new view of diabetic retinopathy: a neurodegenerative disease of the eye. Progress in Neuro-Psychopharmacology and Biological Psychiatry. 2003;27:283–290 doi: 10.1016/S0278-5846(03)00023-X 12657367

[9] Shahidi AM, Sampson GP, Pritchard N, Edwards K, Vagenas D, Russell AW et al … Retinal nerve fibre layer thinning associated with diabetic peripheral neuropathy. Diabet Med 2012; 29:e106–111 doi: 10.1111/j.1464-5491.2012.03588.x 22269030

[10] Srinivasan S, Pritchard N, Vagenas D, Edwards K, Sampson GP, Russell AW et al. Retinal Tissue Thickness is Reduced in Diabetic Peripheral Neuropathy. Curr Eye Res. 2016; 41:1359–1366 doi: 10.3109/02713683.2015.1119855 26928267

[11] El-Fayoumi D, Eldine NMB, Esmael AF, Ghalwash D, Soliman HM. Retinal Nerve Fiber Layer and Ganglion Cell Complex Thicknesses Are Reduced in Children With Type 1 Diabetes With No Evidence of Vascular Retinopathy. Invest Ophthalmol Vis Sci 2016; 57:5355–5360 doi: 10.1167/iovs.16-19988 27737458

[12] van Dijk HW, Verbraak FD, Kok PHB, Stehouwer M, Garvin MK, Sonka M et al. Early neurodegeneration in the retina of type 2 diabetic patients. Invest Ophthalmol Vis Sci 2012; 53:2715–2719 doi: 10.1167/iovs.11-8997 22427582 PMC3366721

[13] Vujosevic S, Midena E. Retinal layers changes in human preclinical and early clinical diabetic retinopathy support early retinal neuronal and Müller cells alterations. J Diabetes Res. 2013:905058. doi: 10.1155/2013/905058 23841106 PMC3694491

[14] Giannini C, Dyck PJ. Basement membrane reduplication and pericyte degeneration precede development of diabetic polyneuropathy and are associated with its severity. Annals of Neurology 1995; 37:498–504 doi: 10.1002/ana.410370412 7717686

[15] Cameron NE, Eaton SEM, Cotter MA, Tesfaye S. Vascular factors and metabolic interactions in the pathogenesis of diabetic neuropathy. Diabetologia 2001; 44:1973–1988 doi: 10.1007/s001250100001 11719828

[16] Nathan DM. The pathophysiology of diabetic complications: how much does the glucose hypothesis explain? Ann Intern Med 1996;124:86–89 doi: 10.7326/0003-4819-124-1_part_2-199601011-00002 8554219

[17] Biallosterski C, van Velthoven ME, Michels RP, Schlingemann RO, DeVries H, Verbraak F. Decreased optical–coherence tomography measured pericentral retinal thickness in patients with diabetes mellitus type 1with minimal diabetic retinopathy. Br J Ophthalmol. 2007; 91 (9): 1135–1138 doi: 10.1136/bjo.2006.111534 17383994 PMC1954913

[18] Srinivasan S, Pritchard N, Sampson GP, Edwards K, Vagenas D, Russell AW, et al. Retinal thickness profile of individuals with diabetes. Ophthalmic Physiol Opt 2016; 36(2): 158–166. doi: 10.1111/opo.12263 26690674

[19] Gołębiewska J, Olechowski A, Wysocka-Mincewicz M, Baszyńska-Wilk M, Groszek A, Czeszyk-Piotrowicz A et al. Choroidal Thickness and Ganglion Cell Complex in Pubescent Children with Type 1 Diabetes without Diabetic Retinopathy Analyzed by Spectral Domain Optical Coherence Tomography. J Diabetes Res 2018:5458015 doi: 10.1155/2018/5458015 29850607 PMC5903202

[20] Srinivasan S, Pritchard N, Sampson GP, Edwards K, Vagenas D, Russell AW et al., Focal loss volume of ganglion cell complex in diabetic neuropathy. Clinical and Experimental Optometry 2016; 99:526–534 doi: 10.1111/cxo.12379 27027413

[21] Gołębiewska J, Olechowski A, Wysocka-Mincewicz M, Odrobina D, Baszyńska-Wilk M, Groszek A, et al. Optical coherence tomography angiography vessel density in children with type 1 diabetes. PLoS One 2017; 12:e0186479 doi: 10.1371/journal.pone.0186479 29053718 PMC5650189

[22] Karsidag S, Morali S, Sargin M, Salman S, Karsidag K, Us O. The electrophysiological findings of subclinical neuropathy in patients with recently diagnosed type 1 diabetes mellitus. Diabetes Res Clin Pract. 2005; 67:211–219 doi: 10.1016/j.diabres.2004.07.017 15713353

[23] Hansen S, Ballantyne JP. Axonal dysfunction in the neuropathy of diabetes mellitus: a quantitative electrophysiological study. Journal of Neurology, Neurosurgery & Psychiatry 1977; 40:555–564 doi: 10.1136/jnnp.40.6.555 903770 PMC492761

[24] Lee S-S, Han H-S, Kim H A 5-yr follow-up nerve conduction study for the detection of subclinical diabetic neuropathy in children with newly diagnosed insulin-dependent diabetes mellitus. Pediatr Diabetes 2010; 11:521–528 doi: 10.1111/j.1399-5448.2009.00636.x 20723100

[25] Parano E, Uncini A, De Vivo DC, Lovelace RE. Electrophysiologic correlates of peripheral nervous system maturation in infancy and childhood. J Child Neurol 1993; 8:336–338 doi: 10.1177/088307389300800408 8228028

[26] Matanovic D, Popovic S, Parapid B, Petronic I, Nikolic D. Neurophysiological evaluation in newly diagnosed Diabetes Mellitus type 1. Cent Eur J Med. 2013; 8(4): 503–508

